# N6-methyladenosine RNA is modified in the rat hippocampus following traumatic brain injury with hypothermia treatment

**DOI:** 10.3389/fnins.2023.1069640

**Published:** 2023-02-15

**Authors:** Jin Cheng, Lian Lin, Jiangtao Yu, Xiaolu Zhu, Haoli Ma, Yan Zhao

**Affiliations:** ^1^Emergency Center, Zhongnan Hospital of Wuhan University, Wuhan, China; ^2^Hubei Clinical Research Center for Emergency and Resuscitation, Zhongnan Hospital of Wuhan University, Wuhan, China; ^3^Department of Emergency, Gansu Provincial People’s Hospital, Lanzhou, China; ^4^Department of Biological Repositories, Zhongnan Hospital of Wuhan University, Wuhan, China

**Keywords:** traumatic brain injury, m6A methylation, hypothermia, rat hippocampus, epigenetic modification

## Abstract

Recent studies have suggested a role for N6-methyladenosine (m6A) modification in neurological diseases. Hypothermia, a commonly used treatment for traumatic brain injury, plays a neuroprotective role by altering m6A modifications. In this study, methylated RNA immunoprecipitation sequencing (MeRIP-Seq) was applied to conduct a genome-wide analysis of RNA m6A methylation in the rat hippocampus of Sham and traumatic brain injury (TBI) groups. In addition, we identified the expression of mRNA in the rat hippocampus after TBI with hypothermia treatment. Compared with the Sham group, the sequencing results of the TBI group showed that 951 different m6A peaks and 1226 differentially expressed mRNAs were found. We performed cross-linking analysis of the data of the two groups. The result showed that 92 hyper-methylated genes were upregulated, 13 hyper-methylated genes were downregulated, 25 hypo-methylated genes were upregulated, and 10 hypo-methylated genes were downregulated. Moreover, a total of 758 differential peaks were identified between TBI and hypothermia treatment groups. Among these differential peaks, 173 peaks were altered by TBI and reversed by hypothermia treatment, including Plat, Pdcd5, Rnd3, Sirt1, Plaur, Runx1, Ccr1, Marveld1, Lmnb2, and Chd7. We found that hypothermia treatment transformed some aspects of the TBI-induced m6A methylation landscape of the rat hippocampus.

## Introduction

Globally, traumatic brain injury (TBI) is one of the main causes of death and disability (Coronado et al., 2012). It has been reported that in Europe over 7 million people suffer from learning and physical disability induced by TBI (Tagliaferri et al., 2006), and patients diagnosed with severe TBI mortality over a third (Stein et al., 2010). Society and healthcare systems worldwide are heavily burdened by TBI patients’ clinical management and associated socioeconomic problems (Khellaf et al., 2019). Patients with TBI may have a range of functional problems, such as cognitive, sensorimotor, and post-concussive symptoms, the impact and severity of which depend on the position and severity of injury (Blennow et al., 2016; Pavlovic et al., 2019). The TBI can be categorized as (1) closed head, (2) penetrating, and (3) explosive blast TBI, according to the specific physical mechanisms of injury (Bruns and Hauser, 2003). The primary damage caused by the injury depends on the mechanical forces impacting the brain tissues directly. The secondary damage is caused by a cascade of cellular and molecular changes triggered by the primary damage. Furthermore, the secondary damage includes hypoglycemia, hypoxic, and hypotensive forms, which cause high intracranial pressure reducing cerebral blood supply (Andriessen et al., 2010; McGinn and Povlishock, 2016; Dixon, 2017). Over time, studies have used TBI models to show that a certain level of hypothermia is effective in reducing brain edema and improving functional outcomes (Ahmed et al., 2016; Docherty et al., 2018). Evidence has increasingly indicated that the TBI instigates the cascade of brain injury in several ways, while hypothermia has a protective effect against brain injury *via* several mechanisms, including inhibiting the production of lactic acid and energy metabolism in the brain (Zhang et al., 2011), reducing endoplasmic reticulum stress-induced apoptosis (Wang C. et al., 2019), promoting neuronal sprouting (Zhao et al., 2017), and alleviating inflammation caused by an elevated NO, ROS, and inflammatory factors and activated glial cells (Lee et al., 2016; Liu et al., 2016; Truettner et al., 2017). According to the results of multiple preclinical studies, mild to moderate hypothermia at an early stage following focal or diffuse TBI has beneficial histopathological, behavioral, and cognitive outcomes (Yokobori et al., 2011; Yokobori et al., 2013; Jin et al., 2015; Lei et al., 2015).

However, the translation of these research findings into the clinical application has proved controversial. Multiple large multicenter clinical trials have not found hypothermia treatment to have a significant protective effect on the brain (Andriessen et al., 2010; Clifton et al., 2011), probably due to the complex and multifactorial nature of cellular responses, as well as a lack of understanding of TBI. Therefore, there is an urgent need to thoroughly investigate the key aspects of physiology in TBI and to improve the patient’s prognosis.

7-methylguanosine (m7G), m1G, m2G, m6G, 5-methylcytosine (m5C), N1-methyladenosine (m1A), and m6A are common RNA methylation sites (Roundtree et al., 2017; Weng et al., 2018). Recent studies have shown that m6A modifications participate in various basic biological processes, such as controlling mRNA translation, splicing, maturation, stability, regulating stem cell differentiation and self-renewal, and regulating RNA–protein interactions (Tong et al., 2018; Zhou et al., 2018). Most of the m6A modifications are enriched at specific transcript landmarks, especially in stop codons, long internal exons, and 5′ and 3′untranslated regions (UTR) (Dominissini et al., 2012). RNA methyltransferases (writers) including METTL3, METTL14, and their cofactors WTAP, catalyze m6A modification. Demethylases (erasers) including FTO and ALKBH5 can remove m6A modification, and m6A-binding proteins (readers) including YTHDC1-2, YTHDF1-3, and IGF2BP1-3 can regulate interaction with m6A (Lan Q. et al., 2019). Several studies (Wang and Zhao, 2016; Li et al., 2018) have indicated that the m6A is closely associated with neural degenerative diseases and the development of the nervous system, but the relevant roles and mechanisms remain unclear. Recent research involving whole-genome profiling of m6A-tagged transcripts in the hippocampus of a TBI mouse model found that METTL3 was downregulated. Using methylated RNA immunoprecipitation sequencing (MeRIP-Seq), 922 significantly differentially expressed m6A peaks were identified, with 370 upregulated and 552 downregulated (Wang Y. et al., 2019). Our research team previously performed an mRNA m6A methylation profile in a genome wide of rat cortex after TBI by m6A MeRIP-Seq (Yu et al., 2020). We found that the expression levels of METTL14 and FTO were significantly downregulated, and the combination of m6A modifications peaks and mRNA-seq analysis showed that 175 mRNAs were significantly altered both in m6A modification and mRNA expression levels after TBI.

To date, the association between m6A methylation and the protective effect of hypothermia against brain injury has not been clarified. With the aim of exploring the mRNA m6A epigenetic modifications in hypothermia protective effects after TBI, we performed an m6A modification analysis of the rat hippocampus following TBI with and without hypothermia treatment. We identified the expression of mRNA in the rat hippocampus after TBI and analyzed m6A methylation and mRNA expression levels. The overall goal of this research is to uncover new approaches for further investigation of TBI hypothermia therapy.

## Materials and methods

### Animals

Adult male Sprague Dawley rats weighing about 250–300 g were bought from the Charles River Laboratories (Beijing, China). All animals were fed in the Animal Experiment Center of Zhongnan Hospital, Wuhan University. All animals were kept in controlled conditions, with the temperature maintained at 25 ± 2°C, the relative humidity at 50 ± 5%, and a 12:12 h light–dark cycle. Food and water were always offered to the rats but were withheld overnight before surgery. Animal experiments were conducted in accordance with the Guidelines of Animal Care and Use. The study was approved by the Animal Experiment Center of Zhongnan Hospital, Wuhan University. All animals were allocated to the following groups: (1) Sham; (2) TBI; and (3) TBI + Hypo (hypothermia) (*n* = 22 per group).

### Controlled cortical injury animal model

We used a controlled cortical impact (CCI) device (Custom Design & Fabrication, USA) to build the animal TBI model as described previously (Osier and Dixon, 2016; Siebold et al., 2018). Rats were anesthetized using an intraperitoneal injection of 5% phenobarbital (50 mg/kg). The skull was exposed *via* a midline incision on the scalp; then, a 5-mm diameter bone window was drilled over the right cerebral hemisphere between the bregma and lambda. The rat was placed on the CCI device with an impact depth of 2 mm, a velocity of 5 m/s, and an impact dwell time of 200 ms, and the impactor tip was used to impact the center of the craniectomy. After impact, the bone was fixed using bone wax and absorbable sutures were used to close the incision. Sham group rats were subjected to the same anesthetic and surgical procedures as those in the TBI group without being subjected to cortical injury. Animals that experienced CCI or sham surgery were held at normothermia or hypothermia for 6 h.

### Temperature manipulations

We referred to the temperature manipulation of the previous research (Cheng et al., 2018). In general, 30 min after completion of sham surgery or CCI, the temperature was manipulated in the TBI + Hypo group. A temperature controller (BP-2010A, Softron, Tokyo, Japan) was used to continuously monitor core body temperature using rectal temperature probes. The back of the prone rat was covered with an ice blanket until the body temperature reached 32 ± 0.5°C and then intermittently used an ice blanket to maintain the temperature of rats at 32 ± 0.5°C. In accordance with the previous research (Silasi and Colbourne, 2011; Jin et al., 2016) and the American Association of Neurological Surgeons Guidelines (Marion et al., 1997), the hypothermia therapy temperature was set at 32°C. After receiving the hypothermia treatment for 6h, the animals were rewarmed over a 1-h period to baseline temperature (37 ± 0.5°C) using an infrared lamp and a heating blanket. TBI group and Sham group animals did not receive hypothermia treatment and were kept at normal baseline temperature after sham surgery or injury.

### Hematoxylin and eosin (HE) staining

One day after TBI, rats (*n* = 3 per group) were anesthetized and a thoracotomy was performed to expose the heart area. A 4% solution of buffered saline and paraformaldehyde (pH 7.2–7.4) was injected to flush the blood and fix the tissue. After full perfusion, the whole brain was extracted and then placed in 4% paraformaldehyde maintained at 4°C overnight. After paraffin embedding, the brain was cut into three 2-mm-thick coronal slices covering the whole damaged area. The sections were then stained with HE (Baso, Wuhan, China) following the conventional method (Tian et al., 2020). After the sections are dewaxed, they are stained with hematoxylin solution for 5 min and then washed with water for 15 min. Then, the slides were stained with eosin solution for 2 min, washed again, and dehydrated with ethanol. A mounting medium was applied once the slides were dried, and the slides were cover-slipped and photographed under a light microscope. Tissue loss area calculations were performed using Image J software.

### Behavioral testing: mNSS and MWM

The modified neurological severity score (mNSS) was evaluated 1 day after injury (*n* = 5 per group). The mNSS is a comprehensive test to evaluate neurological function deficits in rats, including motor, sensory, balance, and reflex assessment, with maximum scores of 6, 2, 6, and 4, respectively (Gold et al., 2013). Total scores of 13–18 indicate severe injury, 7–12 moderate injury, and 1–6 mild injury. Morris water maze (MWM) was used to assess the spatial learning and memory ability of rats at post-injury day 2 (*n* = 5 in each group). As previously described (Vorhees and Williams, 2006), rats were placed on a circular tank containing opaque water from the four water entry points to train them to find a submerged platform, undergoing four trials every day for 5 consecutive days. The incubation period is the time from entering the water to reaching the platform. After 5 days of trials, the escape latency time of each rat was recorded from entering the water to reaching the platform.

### Methylated RNA immunoprecipitation sequencing (MeRIP-Seq)

N6-methyladenosine methylation levels in RNA were measured *via* MeRIP-Seq. At 24 h after CCI, animals were anesthetized and hemispheres were carefully dissected and stored in liquid nitrogen. Three biological replicate samples were modeled in the Sham group, the TBI group, and the TBI + Hypo group, and four hippocampi were collected per group. The total RNA was isolated from the hippocampus of each rat using TRIzol reagent (Invitrogen, Carlsbad, CA, USA), according to the manufacturer’s protocol. Over 200 μg total RNA was collected per sample, and the poly (A) mRNA was isolated using poly-T oligo attached magnetic beads (Invitrogen, USA). The poly (A) mRNA was then fragmented into ∼100-nt-long oligonucleotides using divalent cations at a high temperature, and cleaved RNA fragments were placed into the m6A-specific antibody (No. 202003, Synaptic Systems, Germany) in IP buffer (50 mM Tris-Hcl, 750 mM NaCl, and 0.5% IGEPAL CA-630) with BSA (0.5 μg/μl) for 2 h at 4°C. The mixture was incubated by protein-A beads and eluted by buffer (1 × IP buffer and 6.7 mM m6A). The eluted m6A-containing fragments (IP) and untreated input control fragments were used to construct final cDNA libraries. The mean insert magnitude for the paired-end libraries was ∼150 bp. Finally, the paired-end 2 × 150 bp sequencing was conducted using an Illumina Nova 6000 platform (OE Biotech Co., Ltd., Shanghai, China).

### Data processing and bioinformatics analyses

First, Trimmomatic software was used to process the raw data (raw reads) (Bolger et al., 2014), to remove low-quality reads and those including ploy-N, and to retain the clean reads. The paired reads from clean data were aligned using BLAST software and NT database^1^ with an e-value of <1e-10 and a coverage of >80%. In addition, ribosomal RNA reads were removed using SortMeRNA software (Kopylova et al., 2012), and the clean reads were mapped to the reference genome using HISAT2 (Kim et al., 2015) with the default parameters. Unique reads with a high mapping quality were reserved.

Guitar (Cui et al., 2016) R package and deepTools (Ramírez et al., 2014) software were used to assess the sequencing data quality of methylated RNA immunoprecipitation. MeTDiff (Cui et al., 2018) software was used with parameters fragment_length 200 bp, peak_cutoff_*p*-value 0.01, and peak_cutoff_FDR 0.05 to identify the m6A-enriched peaks in each m6A-immunoprecipitation sample compared with the corresponding input sample. ChIPseeker was applied to interpret the identified peaks through the intersection with the gene construction (Yu et al., 2015). Gene Ontology (GO) enrichment and Kyoto Encyclopedia of Genes and Genomes (KEGG) pathway enrichment analyses of peaks and differential peaks were performed using R software based on the hypergeometric distribution. Sequence motif was detected using MEME (Bailey et al., 2009) and DREME (Schulz et al., 2012) and annotated using TomTom software. Protein–protein interaction network of mRNA expression and m6A peak was performed with Cytoscape Version 3.7.2 software. The statistical analysis was conducted using SPSS Version 25.0 software (IBM, Armonk, NY, USA).

## Results

### Reduced neurological damage and improved behavioral outcome in TBI after hypothermia

**FIGURE 1:**
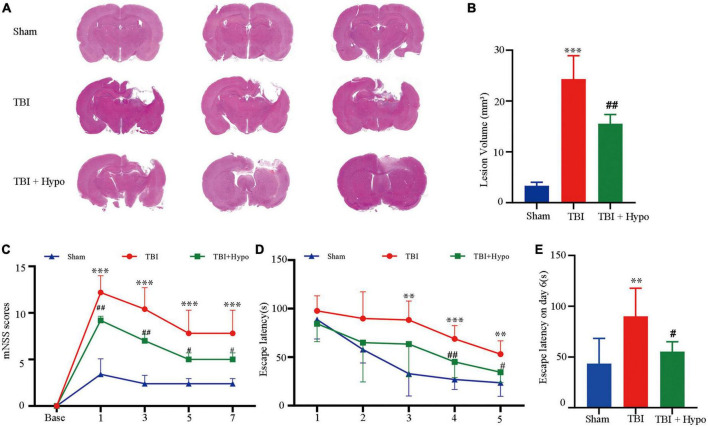
Effects of hypothermia treatment on the neurological damage and behavioral outcomes after the traumatic brain injury (TBI). **(A)** Brain sections stained with HE (*n* = 3 per group). **(B)** Lesion volume calculation from the sections. **(C)** mNSS score on days 1, 3, 5, and 7 post-TBI (*n* = 5 per group). **(D)** Escape latency in MWM test 1–5 days post-TBI. **(E)** Escape latency (*n* = 5 per group) (**p < 0.01, and ***p < 0.001 vs. the Sham group; ^#^*p* < 0.05 and ^##^*p* < 0.01 vs. the TBI group). Error bars represent standard deviation (SD).

The HE staining of brain tissue sections confirmed that hypothermia reduced neurological damage (Figure 1A). Due to cytotoxic or vascular edema, and hydrocephalus induced by trauma, the TBI group showed extensive injury in regions from the cortex to the hippocampus. Quantitative analyses showed volume lesions of 24.33 ± 4.59 mm^3^ in the TBI group and 3.33 ± 0.68 mm^3^ in the Sham group (*p* < 0.001). In agreement with the previous studies, hypothermia significantly decreased CCI-induced damage volumes (15.53 ± 1.80 mm^3^) compared with the TBI group (*p* < 0.01) (Figure 1B).

To test whether hypothermia improved behavioral outcomes, we conducted mNSS and MWM. As shown in Figure 1C, compared with the Sham group, the mNSS score was significantly higher in the TBI group on days 1, 3, 5, and 7 post-TBI (*p* < 0.001 in all groups) and was significantly lower in the TBI + hypo group on these 4 days (*p* < 0.01 on days 1 and 3, *p* < 0.05 on days 5 and 7). Furthermore, we used the MWM test to measure the spatial learning and memory of rats over 5 consecutive days. Compared with the TBI group, the escape latencies were significantly reduced in the TBI + hypo group on days 4 and 5 after TBI (Figure 1D, *p* < 0.01 and *p* < 0.05, respectively). On the test day (day 6), escape latency was significantly longer in the TBI group than in the Sham group (Figure 1E, *p* < 0.01). In contrast, escape latency was significantly shorter in the TBI + hypo group than in the TBI group (Figure 1E, *p* < 0.05).

### M6A modification overview after TBI

**FIGURE 2:**
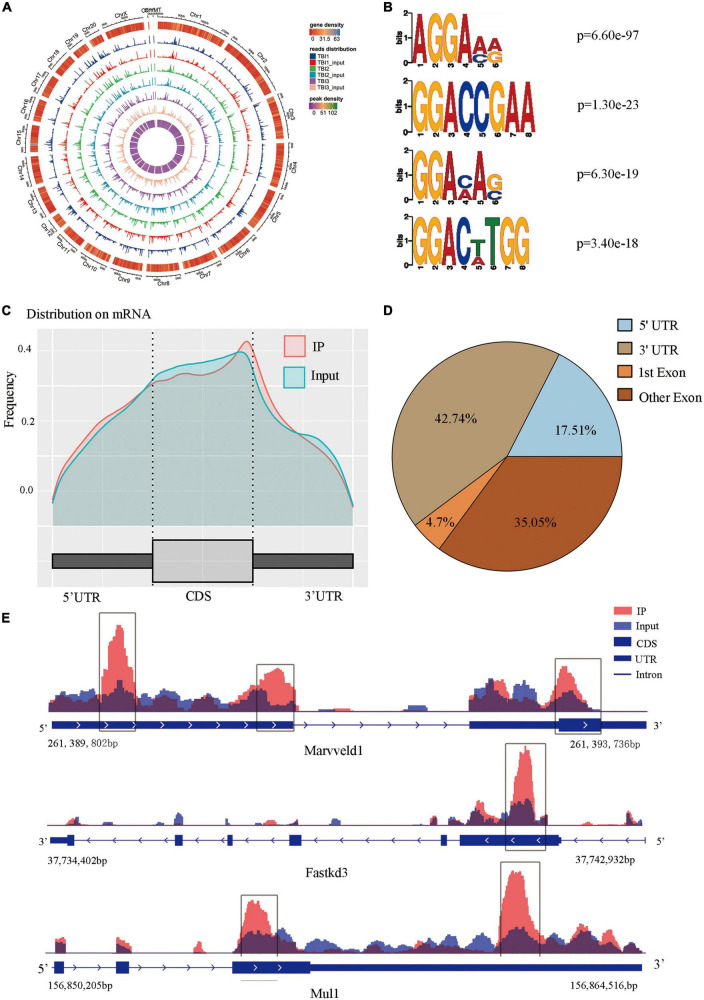
Topological distribution of N6-methyladenosine (m6A) peaks. **(A)** M6A peaks were distributed in different chromosomes. **(B)** The sequence logo represented the deduced consensus motif through the clustering of all enriched m6A peaks. **(C)** M6A peak distribution patterns along the transcript, m6A sites were the most abundant at the 5′terminate and 3′ terminate. **(D)** The sector graph showed the ratio of peaks in each region. **(E)** IGV plot showed directly the peaks in the genes of Marveld1, Fastkd3, and Mul1, the peak positions of Marveld1 were at the CDS region and 5′ UTR, the peak position of Fastkd3 was at the CDS regions, and the peak position of Mul1 was at the CDS and the 3′ UTR regions.

We used m6A MeRIP-Seq to perform a widespread transcriptome m6A-seq analysis and, thus, to observe the m6A methylation modification level in the rat hippocampus after TBI. The detailed raw sequencing data of each group’s MeRIP-Seq samples and input samples are shown in Supplementary Table 1. We found average sequencing data of 11.65 Gb in the MeRIP-Seq samples and 10.25 Gb in the input samples. The averages in the clean data of the MeRIP-Seq samples and input samples of each group are also shown in Supplementary Table 1. Based on the reference genome location information, the percentage of mapped reads ranged from 80.65 to 92.23% (Supplementary Table 2). In addition, the percentage of unique mapped reads in each group ranged from 77.01 to 87.60%. The m6A peaks were identified by comparing MeRIP-Seq sequencing data between IP samples and their corresponding inputs (Figure 2A), and the m6A peaks were distributed across different chromosomes. Moreover, we performed a motif-searching analysis with all m6A peaks and found that consensus GGAC m6A motifs were enriched in RNA, confirming the quality of our data (Figure 2B; Dominissini et al., 2012; Meyer et al., 2012). Analysis of the m6A peak distribution patterns along the transcript showed that the reads of input samples in the CDS region were higher than those of IP samples. We found that m6A sites were the most abundant at the 5′ and 3′ UTR (Figures 2C, D). We chose three significant genes (Marveld1, Fastkd3, and Mul1) to show m6A methylation modification distribution patterns (Figure 2E). The peak positions of Marveld1 were at the CDS region and 5′ UTR, the peak position of Fastkd3 was at the CDS region, and the peak positions of Mul1 were at the CDS and the 3′ UTR regions.

### Significant m6A methylation modification changes by TBI

**FIGURE 3:**
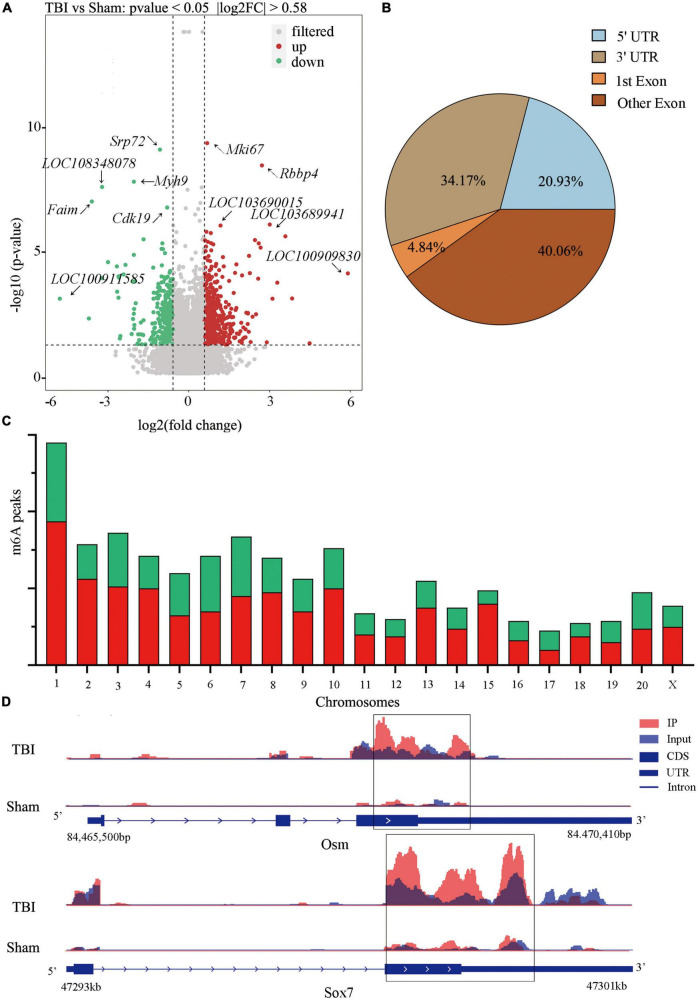
Distribution of significantly changed peaks after traumatic brain injury (TBI). **(A)** The volcano plot showed significantly upregulated and downregulated peaks after TBI. **(B)** The sector graph showed the ratio of significantly changed peaks in each region after TBI. **(C)** Distributions of changed N6-methyladenosine (m6A) peaks in rat chromosomes after TBI. **(D)** Two representative genes that m6A peak was significantly changed after TBI, the m6A level of OSM and Sox7 was significantly upregulated after TBI by 2.38-fold and 2.68-fold, respectively.

We compared alterations in the m6A methylation modification levels of the rat hippocampus between Sham and TBI groups (Supplementary Table 3). In total, 25,486 peaks were identified in the TBI group and 25,094 in the Sham group. The total length of peaks (bp), average length of peaks, median length of peaks, and percentage of the genome of TBI and Sham groups are summarized in Supplementary Table 3. The significantly changed m6A peaks in the hippocampus after TBI are summarized in Supplementary Table 4. We identified 951 significantly changed peaks (*p* < 0.05 and fold change ≥1.5), including 589 upregulated and 362 downregulated peaks (Figure 3A). The top 20 differently expressed m6A peaks after TBI are shown in Table 1. The significantly changed peaks were mainly distributed in the 5′ UTR (20.93%), 3′UTR (34.17%), 1st Exon (4.84%), and another Exon (40.06%) (Figure 3B). Altered m6A peaks were distributed across all chromosomes in the rat hippocampus but were particularly abundant in chr1, chr3, and chr7 (Figure 3C). We chose two representative genes (Osm and Sox7) with significantly changed m6A peaks to show their m6A methylation pattern after TBI (Figure 3D) and found an obvious upregulation of m6A methylation modification levels after TBI by a factor of 2.38 and 2.68, respectively.

**TABLE 1:** Top 20 differently expressed N6-methyladenosine (m6A) peaks after traumatic brain injury (TBI).

Gene	Chromosome	Peak start	Peak end	Lg (*p*-value)	Different log2 (fold change)	Up/Down
Pmm2	Chr10	7068131	7074226	−1.92E-11	−0.843	Down
Mcemp1	Chr12	2213717	2214046	−7.96E-10	−0.798	Down
Erbb3	Chr7	2990648	2990849	−9.50E-10	−0.673	Down
Fbln2	Chr4	122835484	122855515	−1.55E-09	−0.613	Down
Tbcd	Chr10	110699126	110699324	−2.79E-09	−0.778	Down
LOC100911727	Chr1	75260760	75262381	−6.26E-09	−2.04	Down
Mul1	Chr5	156864121	156864270	−1.13E-08	−0.777	Down
Hectd1	Chr6	72552159	72555267	−3.88E-08	−0.7	Down
Ntmt1	Chr3	9643057	9650355	−1.07E-07	−0.662	Down
Hipk1	Chr2	206273985	206274186	−1.51E-07	−0.591	Down
Cbln1	Chr19	20610268	20610668	−59.5	0.585	Up
Ppp1r12b	Chr13	51708294	51709259	−34.7	0.776	Up
Ccdc112	Chr18	40254647	40254796	−32	1.37	Up
Cfap126	Chr13	89492031	89493656	−31.9	0.798	Up
LOC100911068	Chr8	39890890	39891485	−30.8	0.581	Up
Ppfibp2	Chr1	171816230	171820393	−25.6	0.823	Up
Mrvi1	Chr1	175684954	175685154	−25.4	1.18	Up
Far2	Chr4	182563185	182563485	−20.5	0.637	Up
Fam49b	Chr7	104523016	104525438	−20.5	0.656	Up
Rapgef2	Chr2	177867728	177867928	−19.9	0.696	Up

### GO analysis and KEGG pathway analysis of RNA m6A methylation after TBI

**FIGURE 4:**
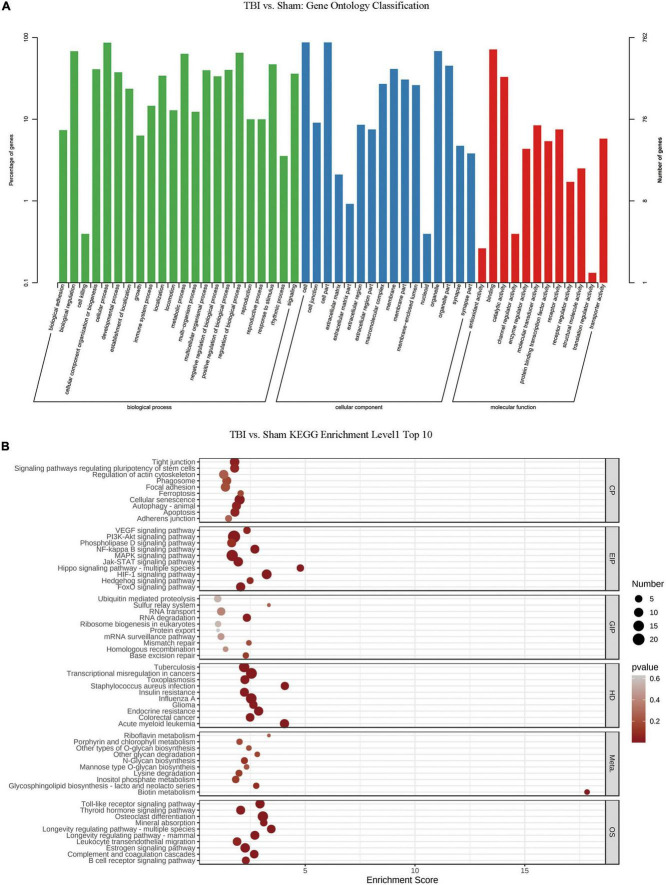
Gene ontology enrichment and Kyoto Encyclopedia of Genes and Genomes pathway analysis of changed N6-methyladenosine (m6A) transcripts. **(A)** Gene ontology classification of the m6A peaks. **(B)** Top 10 enriched pathways of m6A peaks.

We conducted GO and KEGG pathway analyses of significantly changed m6A peaks after TBI. In the biological process (BP) category of GO analysis, we observed that the genes with methylated m6A peaks were significantly enriched in cellular process, biological regulation, regulation of the biological process, metabolic process, response to stimulus, cellular component organization or biogenesis, positive regulation of the biological process, and multicellular organismal process. Significant GO cell component (CC) terms showed that m6A methylations were associated with the cell, cell part, organelle, organelle part, membrane, membrane part, macromolecular complex, and membrane-enclosed lumen. For MF term, these methylations were associated with binding, catalytic, molecular transducer, receptor, transporter, protein-binding transcription factor, enzyme regulator, and structural molecule activities (Figure 4A). KEGG pathway analysis revealed that the pathways affected by significantly changed m6A peaks were significantly associated with cellular senescence, HIF-1 signaling, PI3K-Akt signaling, MAPK signaling, and transcriptional dysregulation in cancers (Figure 4B).

### M6A modification profiles in the hippocampus of post-TBI hypothermia treatment rat model

**FIGURE 5:**
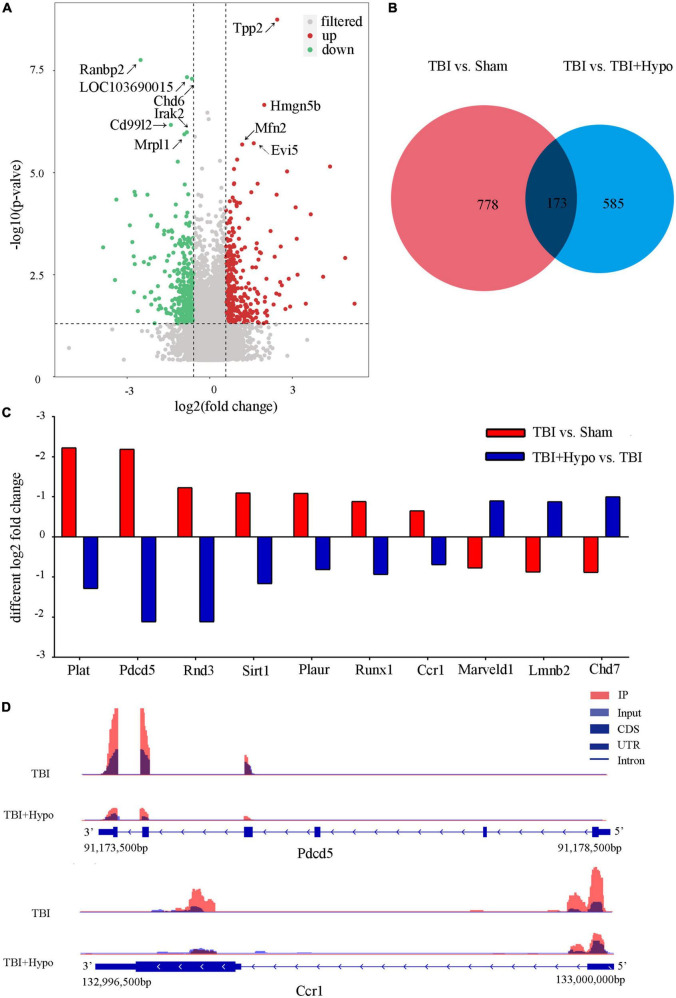
Distribution of significantly changed peaks after post-traumatic brain injury (TBI) hypothermia treatment. **(A)** The volcano plot showed significantly upregulated and downregulated peaks after TBI. **(B)** Venn diagram, TBI vs. sham, and TBI + Hypo vs. TBI showed overlap in differentially expressed peaks. **(C)** Examples of peaks significantly changed after hypothermia treatment. **(D)** Two representative genes, Pdcd5, and Ccr1, the m6A level of Pdcd5 and Ccr1 were upregulated after TBI and reversed by hypothermia treatment.

In order to explore the response of m6A modification to hypothermia, we compared the m6A methylation between rats with TBI and post-TBI hypothermia treatment. We identified 758 significantly changed peaks (*p* < 0.05 and fold change ≥1.5), including 399 significantly upregulated and 359 significantly downregulated (Figure 5A). The significantly changed m6A peaks after hypothermia treatment are summarized in Supplementary Table 5. A total of 951 peaks were modified differently between Sham and TBI groups and 758 peaks between TBI and TBI + Hypo groups. The top 20 differently expressed m6A peaks between the TBI group and TBI+Hypo group are shown in Table 2. Among these differential peaks, 173 were induced by TBI and reversed by hypothermia treatment (Figure 5B). Some peaks were upregulated after TBI and reversed by hypothermia treatment, such as Plat, Pdcd5, Rnd3, Sirt1, Plaur, Runx1, and Ccr1 (Figure 5C). In contrast, expression levels of some peaks were downregulated after TBI and upregulated by hypothermia treatment, such as Marveld1, Lmnb2, and Chd7 (Figure 5C). We chose two genes (Pdcd5 and Ccr1) to show the changes in m6A methylation peaks. The m6A levels of Pdcd5 and Ccr1 were significantly upregulated after TBI and downregulated after hypothermia treatment (Figure 5D).

**TABLE 2:** Top 20 differently expressed N6-methyladenosine (m6A) peaks between the traumatic brain injury (TBI) group and the TBI + Hypo group.

Gene	Chromosome	Peak start	Peak end	Lg (*p*-value)	Diff.log2 (fold change)	Up/Down
Foxc1	Chr17	33950935	33951235	−32.9	0.751	Up
LOC108350796	Chr4	182563666	182563815	−32.1	1.1	Up
Arhgap26	Chr18	32080267	32092383	−28.3	0.62	Up
Hemk1	Chr8	116085462	116090352	−27.9	0.837	Up
Myh6	Chr15	33607906	33610786	−27.5	0.638	Up
Ppm1l	Chr2	166215833	166215983	−25.8	0.848	Up
Tsr2	ChrX	20227301	20227793	−25.1	1.28	Up
Depdc5	Chr14	83197422	83208880	−24.7	0.77	Up
Fam110b	Chr5	18902819	18903020	−24.2	0.878	Up
Ak5	Chr9	10023070	10023265	−24	0.704	Up
Fat2	Chr10	40594683	40594833	−9.79E-15	−1.14	Down
Fat2	Chr10	40583124	40583274	−3.29E-14	−2.01	Down
Fat2	Chr10	40616334	40616584	−5.93E-14	−1.91	Down
Dpp10	Chr13	39431186	39431386	−7.36E-14	−1.08	Down
Fat2	Chr10	40613841	40614092	−1.48E-12	−2.26	Down
LOC108349349	Chr19	275607	276917	−1.80E-09	−1.32	Down
Rnd3	Chr3	36643373	36643569	−4.72E-09	−2.13	Down
Bdnf	Chr3	100770295	100771082	−6.87E-09	−1.38	Down
Frmpd4	ChrX	27554766	27554917	−3.39E-08	−1.18	Down
Tmem121	Chr6	138000755	138000906	−8.96E-08	−2.72	Down

### United analysis of m6A MeRIP-Seq and RNA-seq data

**FIGURE 6:**
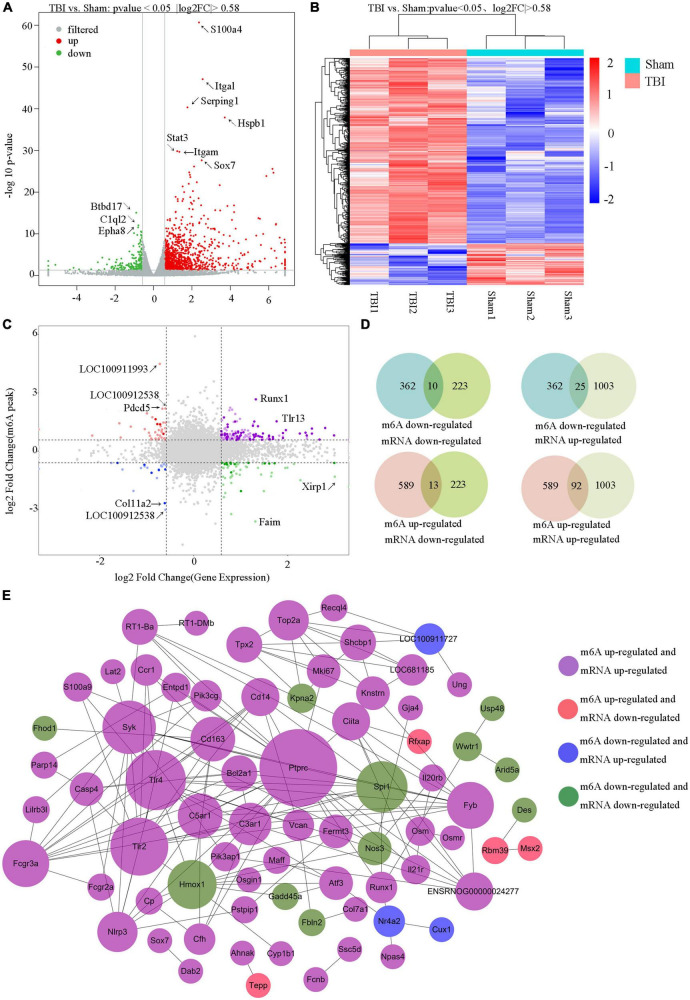
Unite analysis of N6-methyladenosine (m6A) methylation and RNA expression levels. **(A)** The volcano plot shows the significantly upregulated and downregulated mRNA. **(B)** Hierarchical clustering analysis of the differentially expressed mRNAs. **(C,D)** Four quadrant graphs and a Venn diagram showed the relationship between mRNA m6A methylation and its mRNA expression. **(E)** Protein–protein interaction network was performed to show the connection between the proteins encoded by genes that their mRNA expression levels and m6A methylation peaks were both changed significantly.

The RNA-seq data of input samples were used to determine transcriptome profiles of differential expression genes in the rat hippocampus after TBI. We identified significantly expressed genes (*p* < 0.05 and fold change ≥1.5) between the Sham and TBI groups (Supplementary Table 6). We found that 1226 genes were differentially expressed after TBI, including 1003 mRNAs significantly upregulated and 223 mRNAs significantly downregulated (Figure 6A). The top 20 changed genes are shown in Table 3. The gene expression pattern is shown by hierarchical clustering (Figure 6B). The unite analysis results of m6A methylation modification and mRNA expression after TBI are summarized in Supplementary Table 7. We found 140 genes that showed significant changes in mRNA expression levels and m6A methylation peaks, including 10 hypo-methylated with mRNA down-expression, 25 hypo-methylated with mRNA up-expression, 13 hyper-methylated with mRNA down-expression, and 92 hyper-methylated with mRNA up-expression (Figures 6C, D). The link between the proteins encoded by the 140 modified genes is shown using a protein–protein interaction network (Figure 6E).

**TABLE 3:** Top 20 differently expressed genes after traumatic brain injury (TBI).

Gene	Fold change	Log2 fold change	*P*-value	P-adj	Up/Down
S100a4	5.160487026	2.367507228	2.12E-61	3.95E-57	Up
Itgal	5.894128407	2.559278491	8.38E-48	7.81E-44	Up
Serping1	3.406242319	1.768181072	4.84E-41	3.01E-37	Up
Hspb1	13.08855847	3.710234308	1.30E-38	6.04E-35	Up
Stat3	2.349677698	1.232462878	1.52E-30	5.68E-27	Up
Itgam	2.541809871	1.34585612	2.17E-30	6.73E-27	Up
Sox7	5.702346224	2.511555637	2.36E-28	6.27E-25	Up
Pdpn	4.322268276	2.111788621	7.10E-27	1.66E-23	Up
Clec7a	73.37123169	6.197142599	2.69E-26	5.57E-23	Up
Gbp2	3.632253298	1.860864813	1.86E-25	3.46E-22	Up
C1ql2	0.534533023	−0.903649017	8.50E-16	3.86E-13	Down
Epha8	0.577906089	−0.791093025	9.49E-13	2.68E-10	Down
Btbd17	0.572832805	−0.80381398	2.83E-12	7.65E-10	Down
Tril	0.634224896	−0.656933584	2.66E-11	5.70E-09	Down
Plpp3	0.647303246	−0.627486356	3.93E-11	8.23E-09	Down
Htr5b	0.493345443	−1.019329912	6.67E-11	1.35E-08	Down
Lix1	0.634268658	−0.656834041	1.75E-10	3.14E-08	Down
Yjefn3	0.556260714	−0.846166876	1.87E-10	3.26E-08	Down
Cbs	0.647961446	−0.62602012	3.00E-10	4.90E-08	Down
Col11a2	0.652062245	−0.616918406	9.93E-10	1.43E-07	Down

## Discussion

As the most widely distributed and plentiful interior modification of mRNAs, m6A has been a focal point in the area of epitranscriptomics in recent years. M6A modifications play a part in nearly all aspects of physiological behavior, and research has confirmed that m6A modification participates in mRNA splicing, stability, nuclear transport, orientation, translational efficiency activation, and reduced target mRNA stability (Liu et al., 2014; Deng et al., 2018). The scope for m6A modification is extensive in the central nervous system, where it plays important roles in the differentiation of embryonic stem cells, cerebral development, and neurodevelopmental disease (Meyer et al., 2012; Geula et al., 2015). RNA m6A methylation shows heterogeneity in the methylation site and level, as confirmed by m6A immunoprecipitation experiments in mouse cerebellum, hippocampus, and cortex (Chang et al., 2017; Wang Y. et al., 2019). Our research team previously used m6A MeRIP-Seq to measure the m6A methylation modification level of the cortex in a rat TBI model (Yu et al., 2020). In this study, we performed an m6A modification analysis in the rat hippocampus of TBI and post-TBI hypothermia treatment.

We identified 951 significantly changed peaks, including 589 upregulated and 362 downregulated peaks. We performed a motif-searching analysis of all m6A peaks and found consensus GGAC m6A motifs were enriched in RNA, in accordance with the previous reports (Dominissini et al., 2012; Meyer et al., 2012). Interestingly, the modified m6A methylation peaks in TBI are particularly evident in CDS, 3′ UTRs, and 5′ UTRs, again consistent with the previous research (Dominissini et al., 2012). Altered m6A peaks were distributed across all chromosomes in the rat hippocampus after TBI, in line with the m6A peaks variation after TBI in mice (Wang Y. et al., 2019). We demonstrated significant m6A modification in two genes, Osm and Sox7. It is known that oncostatin M (Osm) is one of the interleukins (IL)-6 family of cytokines and plays a broad role in inflammation, cell proliferation, and hematopoiesis (Tanaka and Miyajima, 2003; Chen et al., 2006). Previous research has shown that administering recombinant human Osm to mice before the initiation of ischemia/reperfusion can improve prognosis in mouse models of ischemic stroke by activating the JAK2/STAT3 signaling pathway in neurons (Guo et al., 2015). It has also been shown that Osm treatment significantly diminishes astrocytosis and immune cell infiltration, reduces lesion size, and improves locomotor recovery after mild and severe spinal cord injury (Slaets et al., 2014). We found upregulation of the m6A methylation peak of Osm in the rat hippocampus after TBI; however, the role of Osm m6A methylation in TBI is less well understood and needs further study. As a critical member of the Sox F family, Sox7 has a high mobility group DNA-binding domain. The study demonstrated a new signaling mechanism in the apoptosis of neurons and found Sox7 accelerated neuronal apoptosis by affecting β-catenin activity (Wang et al., 2015). However, the role of Sox7 m6A methylation in TBI remains unclear.

In addition, it has been found in our study that mild hypothermia treatment reversed TBI-induced methylation modification of Pdcd5 and Ccr1. Pdcd5 plays an important role in the process of cell apoptosis, and it was downregulated in many cancers (Choi et al., 2015). The previous reports have shown that the inhibition of Pdcd5 expression in a brain ischemia/reperfusion model improves neurological deficits and cerebral blood flow, reduces the infarct volume, and protects the BBB *via* suppressing the process of neuronal apoptosis and autophagy (Chen et al., 2013; Jiang et al., 2014). However, it has not been proved that the methylation modification of Pdcd5 participates in the treatment of TBI in rats. Ccr1 is involved in the chemotaxis of the nervous system inflammation. Some reports have proved that Ccr1 plays an important role in the inflammatory process of a variety of nervous system diseases (Alzheimer’s disease, multiple sclerosis, and cerebral hemorrhage) (Halks-Miller et al., 2003; Ubogu et al., 2006; Yan et al., 2020). Moreover, it has been reported that the inhibition of Ccr1 reduces BBB damage, brain edema, and neuronal damage and further plays a neuroprotective role in ICH mice (Yan et al., 2022). Methylation modification of PDCD5 and Ccr1 may become a new direction of TBI treatment.

At the cellular level, the damage caused by TBI may occur through four basic mechanisms: inflammatory events, cytotoxicity, calcium-mediated damage, and free radical-induced alterations (Gennarelli and Graham, 1998; Sahuquillo and Vilalta, 2007). Together, these contribute to other lesions, including increased intracranial pressure and brain swelling, apoptosis, perilesional depolarization, and mitochondrial dysfunction. In the present study, GO analysis showed highly enriched m6A methylation for the regulation of biological and metabolic processes, indicating that m6A methylation may play an important role in the process of metabolic alteration after TBI. KEGG pathway enrichment analysis revealed that HIF-1, PI3K-Akt, and MAPK signaling pathways were significantly enriched, implying that m6A modification may involve a range of physiological processes in TBI. The previous study has confirmed that selective inhibition of HIF-1α decreases brain edema, ameliorates neuronal damage, and improves cognitive and motor behavior tests outcomes (Shenaq et al., 2012) and that PI3K-Akt signaling pathway activation improves neurofunctional deficits, preserves blood–brain barrier (BBB) integrity, and leads to restoration of cognitive function after TBI (Wu et al., 2008; Wu et al., 2017). Research has indicated that the MAPK signaling pathway can be activated by TBI and that restraining the MAPK signaling pathway alleviates damage to cognitive functions and lowers the BBB permeability induced by TBI (Lan Y. et al., 2019). However, further research is needed to verify the role of the m6A modification function of these signaling pathways and reveal the regulatory mechanism underlying the phenomenon in TBI.

Microcirculatory disturbances, cerebral blood flow reduction, and deterioration in the homeostasis of cerebral metabolism are early changes in post-TBI. Early studies found that the neuroprotective effect of hypothermia on the brain may be due to a decrease in the cerebral metabolic rates of glucose and oxygen. The use of therapeutic hypothermia following TBI has been widely studied, but the therapeutic mechanisms of hypothermia TBI-induced brain injury have not been clearly understood. Since the brain is rich in m6A, the present study explored the m6A landscape in the rat hippocampus in TBI with and without hypothermia treatment in a rat model. Compared to the TBI group, we identified 758 significantly changed peaks, 399 being upregulated and 359 downregulated. Among these differential peaks, 173 were induced by TBI and were changed by hypothermia treatment. These peaks may be connected to the mechanism underpinning hypothermia’s protective effect on the brain. Among the 173 peaks, many are known to be associated with neurodegenerative diseases. Peaks such as Plat, Pdcd5, Rnd3, Sirt1, Plaur, Runx1, and Ccr1 were upregulated after TBI, and this change was reversed by hypothermia treatment; peaks such as Marveld1, Lmnb2, and Chd7 were significantly downregulated by TBI, and this was reversed by hypothermia treatment. Many of these peaks have been studied and shown to be associated with central nervous system injury. The expression of tissue-type plasminogen activator (t-PA) was encoded by the gene Plat. The tissue-released t-PA can efficiently eliminate the fibrin deposition at the luminal side of an intact vascular endothelium, playing an important role in reducing tissue damage (Kruithof and Dunoyer-Geindre, 2014). Ascl1, a proneural factor expressed in the embryonic cortex, reportedly increases neuronal transfer by adjusting Rnd3, a Rho protein with a role in forwarding extracellular signals to the actin cytoskeleton (Pacary et al., 2011). Recent research found a decreased rate of brain apoptosis in Rnd3-knockout mice and that the regulation of central nervous system apoptosis, *via* the RND3-NF-κB P65 signaling pathway, may be an alternative approach for the treatment of neurodegenerative diseases (Dong et al., 2021). Plaur-deficient mice show impaired somatomotor recovery and emotional learning compared with wild-type mice after TBI (Bolkvadze et al., 2016). Suppression of CCR1 activation by the CCR1/TPR1/ERK1/2 signaling pathway in an intracerebral hemorrhage mouse model can attenuate neuroinflammation, hence decreasing brain edema and improving cognitive functions (Yan et al., 2020). Marveld11 plays an important role in mouse cerebrum development, and a lack of Marveld11 results in an abnormality of motor and cognition functions (Liu et al., 2018). As a major regulator of neurogenesis in the mammalian cerebrum, chromodomain-helicase-DNA-binding protein 7 (Chd7) plays a vital role in the activation of the neuronal differentiation program in neural stem cells (Feng et al., 2013).

M6A-modification is the most abundant and widespread modification of mRNA; therefore, the level of m6A-modification is closely related to RNA nuclear splicing, export, stability, trafficking, and translation efficiency. M6A reader proteins can recognize m6A modification sites such as YTHDF2/3 and can regulate post-transcriptional modification by combining with different complexes (Yen and Chen, 2021). According to the analysis of m6A methylation modification and mRNA expression after TBI, our study identified many genes with significantly modified mRNA expression levels and m6A methylation peaks. The results of our study may form a basis for future research on the function of m6A methylation in traumatic brain injury.

## Conclusion

By using methylated RNA immunoprecipitation sequencing, we measured the hippocampus m6A methylation level in the rat after TBI with and without hypothermia treatment. We found hypothermia treatment transformed some aspects of the TBI-induced m6A methylation landscape of the rat hippocampus. In total, 951 significantly changed peaks were identified between TBI and Sham groups, and 758 significantly changed peaks were identified between TBI and TBI + Hypo groups. We found that the epigenetic modifications of RNA may have functions in the protective effect of hypothermia after TBI. Using a united analysis of m6A methylation modification and mRNA expression, our study identified 140 genes with significantly modified mRNA expression levels and m6A methylation peaks, which may provide enlightenment and guidance for future research on the function of m6A methylation in traumatic brain injury.

## Data availability statement

All of the datasets in this study can be obtained in the public database SRA (accession: PRJNA892082).

## Ethics statement

All animal experimental procedures were approved by the Institutional Animal Care and Use Committee of Wuhan University (ZN202192).

## Author contributions

YZ and HM conceived and designed the research plans and supervised and fixed the writing. JC, LL, JY, and XZ established the TBI model and isolated RNA samples from hippocampus tissues. JC and LL completed the data processing, normalization, bioinformatics analyses, and wrote the manuscript with contributions from all the authors. All authors have read and approved the manuscript.

## Footnotes

1
ftp://ftp.ncbi.nih.gov/blast/db
